# Differential TET1/2/3 Protein Expression in Circulating Leukocytes of Statin-Treated Patients with HFrEF

**DOI:** 10.3390/cimb48050467

**Published:** 2026-04-30

**Authors:** Anna Wołowiec, Łukasz Wołowiec, Albert Jaśniak, Grzegorz Grześk, Jacek Budzyński, Joanna Osiak-Gwiazdowska, Paulina Jakubowska, Paweł Gordon, Mariusz Kozakiewicz

**Affiliations:** 1 Department of Geriatrics, Division of Biochemistry and Biogerontology, Faculty of Health Sciences, Ludwik Rydygier Collegium Medicum in Bydgoszcz, Nicolaus Copernicus University in Toruń, 85-067 Bydgoszcz, Poland; markoz@cm.umk.pl; 2 Department of Cardiology and Clinical Pharmacology, Faculty of Health Sciences, Ludwik Rydygier Collegium Medicum in Bydgoszcz, Nicolaus Copernicus University in Toruń, 85-067 Bydgoszcz, Poland; lukasz.wolowiec@cm.umk.pl (Ł.W.); albert.jasniak@gmail.com (A.J.); g.grzesk@cm.umk.pl (G.G.); joanna.osiak@doktorant.umk.pl (J.O.-G.); 3 Department of Vascular and Internal Diseases, Faculty of Health Sciences, Ludwik Rydygier Collegium Medicum in Bydgoszcz, Nicolaus Copernicus University in Toruń, 85-067 Bydgoszcz, Poland; jb112233@cm.umk.pl; 4 Department of Cardiology and Cardiac Surgery, 10th Military Clinical Hospital, 85-067 Bydgoszcz, Poland; paujaku@wp.pl (P.J.); pawelgo80@gmail.com (P.G.)

**Keywords:** statins, TET enzymes, epigenome, DNA methylation, heart failure with reduced ejection fraction, epigenetics, flow cytometry, immune cells, leukocytes

## Abstract

Epigenetic mechanisms, including DNA methylation and hydroxymethylation, contribute to inflammation, cardiac remodelling and progression of heart failure. Ten–Eleven Translocation (TET) dioxygenases are key regulators of these processes, but the impact of statins on TET proteins in human heart failure is not well characterised. We investigated how statin therapy relates to TET1, TET2 and TET3 expression in circulating immune cells in heart failure with reduced ejection fraction (HFrEF). In this cross-sectional study, 106 patients with HFrEF were enrolled; 84 were receiving statins and 22 were not. Intracellular TET1/2/3 protein levels were measured by multiparameter flow cytometry in granulocytes, monocytes and lymphocytes, and clinical and laboratory characteristics were compared between groups. Statin-treated patients had lower NT-proBNP concentrations and lower neutrophil, lymphocyte and monocyte counts, and more often received guideline-directed medical therapy. Statin therapy was associated with a distinct TET expression profile, characterised by higher TET1 and TET3 indices in monocytes and lymphocytes and lower TET2 indices in granulocytes and monocytes. This pattern is compatible with a distinct immune-cell TET expression profile aligned with the anti-inflammatory and reparative profile attributed to statins, and the course of disease. These associations do not establish causality and require prospective validation. TET proteins may form part of an epigenetic signature associated with statin treatment in heart failure and warrant further study as potential biomarkers in larger, prospective cohorts.

## 1. Introduction

Ten–Eleven Translocation (TET) enzymes play an important role in epigenetic regulation by catalyzing the oxidation of 5-methylcytosine (5mC) to 5-hydroxymethylcytosine (5hmC), thereby influencing chromatin accessibility and gene expression [1]. All three human paralogs—TET1, TET2, and TET3—exhibit 5mC dioxygenase activity, yet differ in their domain architecture and tissue-specific expression patterns [2]. Epigenetic modifications influence a range of pathophysiological processes, including inflammation, oxidative stress, cardiac remodeling, and vascular endothelial function [2]. Their relevance in the cardiovascular system and the pathogenesis of HF has garnered increasing research interest in recent years, due to the correlation between distinct epigenetic alterations and cardiovascular diseases (CVDs) [3,4]. Analysis of these modifications provides molecular insights into the cardiovascular system, from its development to its ongoing function, and may elucidate the complex mechanisms underlying HF. A number of hypermethylated genes and microRNAs, such as *HEY2*, *MSR1*, *MYOM3*, *COX17*, and *miR-24-1*, as well as hypomethylated targets—including *CTGF*, *MMP2*, and *miR-155*—have been identified in interventricular septal tissues of patients with HF, underscoring the role of nucleic acid methylation in regulating HF-associated genes [5].

These processes align with the pleiotropic actions of statins [6], and in the present pilot study, we assessed for the first time TET1/2/3 protein expression by flow cytometry in patients with chronic HFrEF receiving statin therapy.

### 1.1. The Role of Individual TET Proteins in the Cardiovascular System

#### 1.1.1. TET1

TET1 is involved in the regulation of genes associated with the differentiation of vascular smooth muscle cells and cardiomyocytes. Its enzymatic activity contributes to the maintenance of endothelial homeostasis as well as the differentiation, proliferation, and survival of cardiomyocytes [7,8]. Studies suggest that reduced TET1 expression in cardiomyocytes may lead to the accumulation of 5mC in the promoter regions of genes involved in repair processes. This stabilization of adverse epigenetic patterns contributes to maladaptive cardiac remodeling [9,10,11]. TET1 also modulates the expression of genes involved in oxidative stress responses and enhances cellular tolerance to hypoxia by stabilizing hypoxia-inducible factor alpha (HIF-α) proteins [12]. Furthermore, it has been implicated in obesity-related signaling pathways [13]. Notably, TET1 regulates hypoxia-induced endothelial-to-mesenchymal transition (EndMT), a key process in the initiation and progression of various CVDs [14,15,16].

#### 1.1.2. TET2

TET2 is the most extensively studied member of the TET family in the cardiovascular system, with prominent roles in cardiac development, endothelial homeostasis, and regulation of inflammatory responses in haematopoietic cells.

Immunohistochemical analysis of TET1 and TET2 expression at comparable developmental stages revealed robust TET1 expression in the myocardium, while TET2 showed abundant expression across all three layers of the heart wall, including the myocardium, epicardium, and endocardium [17]. A reduction in endothelial TET2 levels has been shown to trigger EndMT, whereas TET2 overexpression inhibits this process [18].

TET2 modulates fibrotic processes, anti-inflammatory and antithrombotic gene expression, and cell survival programmes; its deficiency promotes fibrosis, myocardial stiffness, and accelerated atherogenesis [19].

In animal models, loss of TET2 function in hematopoietic cells enhances inflammatory responses, accelerates atherosclerotic plaque progression, and suggests a causal role for clonal hematopoiesis in cardiovascular disease development [20,21,22,23,24].

Notably, homozygous TET2 mutations expand more rapidly than heterozygous ones and exhibit more severe pathological phenotypes, indicating a dose-dependent effect [20,24,25]. Clonal hematopoiesis resulting from inactivating TET2 mutations is associated with an increased risk and significantly worse prognosis in patients with HF, regardless of its etiology [25,26,27], acute coronary syndrome [28], and those undergoing transcatheter aortic valve implantation [29].

#### 1.1.3. TET3

TET3, similarly to other members of the TET protein family, regulates transcription specific to cardiac embryogenesis by influencing cardiomyocyte differentiation. A deficiency in TET3 has been shown to result in cardiac malformations, as demonstrated in animal models [17]. In a murine model, the deletion of both TET2 and TET3 (TET2/3-DKO) led to ventricular non-compaction cardiomyopathy and embryonic lethality. Impaired DNA demethylation and reduced chromatin accessibility in TET2/3-DKO mice weakened the binding of Ying Yang 1 (YY1) to its genomic targets, thereby disrupting higher-order chromatin organization at key genes involved in heart development [17]. TET3 also plays a regulatory role in the expression of genes associated with endothelial cell proliferation and migration, extracellular matrix protein synthesis, and pathways related to fibrosis and inflammation [30,31,32]. TET3 has been implicated in vascular repair processes and in the regulation of vascular smooth muscle cell proliferation and apoptosis in coronary artery disease [33]. Its activity in inflammatory monocytes has been associated with the inhibition of post-infarction cardiac remodeling [34], and reduced TET3 expression has also been observed in acute Stanford type A aortic dissection [35]. Recent evidence indicates that loss of TET3 in cardiac fibroblasts promotes DNA damage and fibroblast proliferation through dysregulation of DNA repair pathways, and reduced TET3 expression correlates negatively with fibrosis markers in human cardiac tissue [30]. While TET proteins play critical roles in cardiac tissue, their expression in circulating immune cells—which infiltrate the myocardium and actively participate in cardiac inflammation and remodelling—provides a clinically accessible window into the epigenetic state of the immune-cardiac axis.

### 1.2. The Role of Statins in Epigenetic Processes

Recent scientific studies suggest that epigenetic effects associated with statin exposure may mediate some of their pleiotropic actions [6,36,37,38]. Statins have been reported to promote increased histone acetylation and reduced DNA methylation in gene promoter regions, including via inhibition of histone deacetylases (HDACs, “erasers”) and DNA methyltransferases (DNMTs, “writers”). These epigenetic modifications enhance chromatin accessibility, thereby facilitating the transcription of genes involved in cardiovascular function. This effect has been attributed, in part, to inhibition of specific HDACs reported with statin exposure, including HDAC1, HDAC2, HDAC6, HDAC7, and HDAC9 [6]. Statins have also been shown to influence histone methylation, particularly at H3K9 and H3K27, thereby promoting a euchromatic phenotype relevant to the initiation of repair processes following myocardial ischemia [6]. Several studies also report that statin therapy affects both blood lipid levels and DNA methylation patterns. For instance, a significant correlation has been observed between triglyceride levels and the methylation status of the CpG sites cg27243685 and cg17901584 during statin treatment [37].

Available studies indicate that blood lipid levels and DNA methylation patterns undergo changes during statin pharmacotherapy. In the All New Diabetics in Scania (ANDIS) study, an association was found between statin use and epigenetic changes in patients newly diagnosed with type 2 diabetes [38]. Statins generally act as demethylating agents, although the biological consequences of this effect may vary depending on the disease context [36]. Atorvastatin, simvastatin, and pravastatin have been shown to reverse subtelomeric methylation in diabetes mellitus [39]. In a colorectal cancer cell line, lovastatin was found to reverse the silencing of tumor suppressor genes, including those encoding growth factors such as bone morphogenetic protein 2 (BMP2), in a time- and dose-dependent manner. The demethylating properties of lovastatin counteracted the suppressive effect caused by hypermethylation of the BMP2 promoter region [40]. Conversely, atorvastatin has been reported to increase DNMT activity in the human hepatocellular carcinoma cell line HepG2 [41]. Furthermore, demethylation processes appear to underlie some of the immunomodulatory properties of statins. For example, in the presence of TGF-β, simvastatin enhanced forkhead box P3 (Foxp3) transcription in mouse regulatory T cells (Tregs) through demethylation of the Foxp3 promoter [36,42].

Taken together, these data establish that: (i) statins modulate DNA methylation through inhibition of DNMTs [36,37,38,39]; (ii) TET enzymes catalyse the opposing demethylation reaction and are thus functionally coupled to the methylation machinery [1]; and (iii) TET2 alterations in circulating hematopoietic cells are clinically relevant in HFrEF [25,26,27]. Whether statin therapy is associated with altered TET protein expression in circulating immune cells of HFrEF patients has not been investigated. We addressed this question in the present cross-sectional pilot study.

**Figure 1:**
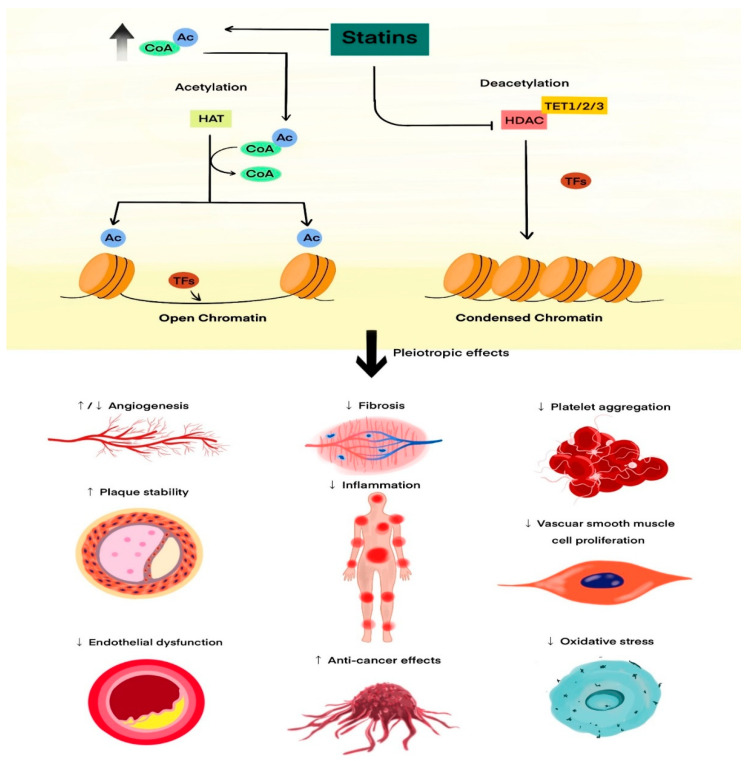
Proposed epigenetic mechanism underlying the pleiotropic effects of statins. Statins inhibit HMG-CoA reductase, reducing not only cholesterol synthesis but also the availability of isoprenoid intermediates (farnesyl pyrophosphate and geranylgeranyl pyrophosphate), which are required for post-translational prenylation of small GTPases (Ras, Rho, Rab). Depletion of these intermediates attenuates NF-κB and AP-1 signalling, dampening pro-inflammatory gene transcription [43]. Statins have been reported to inhibit selected histone deacetylases (HDAC1/2/6/7/9) [6] and DNA methyltransferases (DNMTs) [36], thereby promoting histone acetylation and DNA demethylation at promoter regions of cardioprotective and anti-inflammatory genes. These combined actions shift chromatin towards a more accessible (euchromatic) state, facilitating transcription of genes involved in endothelial homeostasis, antioxidant defence, immune modulation, and tissue repair. The net result may be the epigenetic reprogramming of immune and vascular cells that may underlie the clinically observed pleiotropic benefits of statins beyond LDL-cholesterol lowering. Abbreviations: AcCoA—acetyl coenzyme A; Ac—acetyl group; CoA—coenzyme A; HAT—histone acetyltransferase; HDAC—histone deacetylase; DNMTs—DNA methyltransferases; TFs—transcription factors; HMG-CoA—3-hydroxy-3-methylglutaryl coenzyme A; TET1/2/3—Ten–Eleven Translocation methylcytosine dioxygenase 1/2/3. Arrows indicate proposed stimulatory or inhibitory relationships between statin exposure, epigenetic regulators and downstream anti-inflammatory/cardioprotective pathways.

A potential epigenetic mechanism of the pleiotropic effects of statins is presented in Figure 1.

## 2. Materials and Methods

### 2.1. Study Population

This was a cross-sectional observational study including 106 patients with chronic heart failure with reduced ejection fraction (HFrEF). Of these, 84 patients were receiving statin therapy, and 22 were not receiving statins at the time of blood sampling. Among statin-treated patients (*n* = 84), 54 (64.3%) received atorvastatin, 29 (34.5%) rosuvastatin, and 1 (1.2%) simvastatin. Statin doses were prescribed according to current heart failure and dyslipidaemia guidelines at the discretion of the treating cardiologist. The significantly higher prevalence of diagnosed dyslipidaemia in the statin-treated group (80.9% vs. 18.2%; *p* < 0.001; Table 1) reflects the expected confounding by indication, as statins are preferentially prescribed to patients with this comorbidity. All statin-treated patients had been receiving continuous statin therapy for a minimum of 6 months prior to blood sampling, with a stable statin type and dose for at least 3 months before blood collection. Patients were recruited from the Department of Cardiology and Clinical Pharmacology, Faculty of Health Sciences, Ludwik Rydygier Collegium Medicum in Bydgoszcz, Nicolaus Copernicus University in Toruń (Biziel University Hospital in Bydgoszcz), and from the Department of Cardiology and Cardiac Surgery, 10th Military Clinical Hospital in Bydgoszcz. The diagnosis of HFrEF was based on the criteria of the European Society of Cardiology (ESC). Patients received guideline-directed medical therapy for HFrEF in accordance with ESC recommendations [44]. Peripheral venous blood was collected during routine clinical assessment. All procedures were performed in accordance with the Declaration of Helsinki. The study protocol was approved by the Local Bioethics Committee of the Collegium Medicum in Bydgoszcz, Nicolaus Copernicus University in Toruń (approval no. KB 555/2021). Each patient provided written informed consent after receiving detailed information about the purpose and scope of the study. Inclusion criteria were age ≥ 18 years, chronic heart failure diagnosed according to ESC guidelines, New York Heart Association (NYHA) class II–IV, and left ventricular ejection fraction (LVEF) ≤ 40% assessed during the index hospitalization or within the preceding 6 months. Exclusion criteria were sepsis or shock of any aetiology on admission, acute coronary syndrome, recent (<3 months) myocardial infarction or stroke, active malignancy, autoimmune disease, impaired liver function (international normalized ratio without oral anticoagulation >1.5, total bilirubin > 1.5 mg%, or alanine aminotransferase > 3-fold the upper limit of normal), systemic corticosteroid therapy, decompensated diabetes mellitus requiring intravenous insulin infusion, chronic inflammatory bowel disease, or recent (<3 months) surgery.

### 2.2. Antibody Selection and Optimization

Because no commercially validated antibodies against TET proteins (Tet1, Tet2, Tet3) suitable for direct flow cytometric detection were available, an indirect two-step immunofluorescent staining protocol was applied. Unconjugated primary antibodies were followed by species-specific fluorochrome-conjugated secondary antibodies. Both antibody types were titrated to determine optimal working concentrations, ensuring maximal signal-to-noise ratio and minimal nonspecific binding, according to recommendations by Uhlen et al. [45].

Primary antibodies included rabbit polyclonal anti-Tet1 (Cat. No. ab121587, Abcam Cambridge, UK), goat polyclonal anti-Tet2 (Cat. No. ab99432, Abcam), and rabbit polyclonal anti-Tet3 (Cat. No. ab139311, Abcam). Corresponding secondary antibodies were: donkey F(ab’)_2_ anti-rabbit IgG H&L conjugated with Alexa Fluor^®^ 488 (Cat. No. ab181346, Abcam) and rabbit F(ab’)_2_ anti-goat IgG H&L conjugated with Alexa Fluor^®^ 647 (Cat. No. ab169347, Abcam).

Secondary antibody titration was performed first, and a 1:1000 dilution was found optimal. Subsequently, the primary antibodies were titrated, establishing 1 µL per test as the optimal volume. All staining steps were carried out using Perm/Wash Buffer from the BD Cytofix/Cytoperm™ kit (BD Biosciences).

### 2.3. Sample Preparation

Peripheral blood was collected into EDTA tubes containing TransFix™ reagent (Cytomark, Buckingham, UK) to stabilize surface antigens [46]. Before staining, leukocytes were enumerated using a LUNA-II Automated Cell Counter (Logos Biosystems, Anyang, South Korea) to ensure consistent cell-to-antibody ratios. For each test, 100,000 cells were transferred into a 5 mL round-bottom tube and adjusted to a final volume of 100 µL with phosphate-buffered saline (PBS).

The following fluorochrome-conjugated monoclonal antibodies (BD Biosciences, Franklin Lakes, NJ, USA) were used for surface staining: PE-anti-human CD56/CD16, PerCP-anti-human CD14, APC-H7-anti-human CD19, BV421-anti-human CD45, and V500-anti-human CD3. Staining was performed at room temperature in the dark for 30 min [47]. This 7-marker panel enabled identification of major leukocyte subsets: granulocytes (CD45dim/SSChigh), monocytes (CD45bright/SSCintermediate/CD14+), and lymphocytes (CD45bright/SSClow), with additional lineage markers (CD3, CD19, CD56/CD16) confirming population identity. Intracellular TET protein detection was performed using unconjugated primary antibodies followed by species-specific secondary antibodies conjugated to Alexa Fluor 488 (TET1, TET3) or Alexa Fluor 647 (TET2), as detailed in Section 2.2 and Section 2.4.

Erythrocytes were lysed using 2 mL of FACS Lysing Solution (BD Biosciences) and incubated for 15 min. After two washing steps with PBS and centrifugation (500× *g*, 5 min), cells were fixed and permeabilized in 500 µL of BD Cytofix/Cytoperm™ solution for 20 min in the dark. Cells were then washed with Perm/Wash Buffer and incubated for 10 min before primary antibody application [48].

### 2.4. Intracellular Staining

Primary antibodies against Tet1, Tet2, or Tet3 were added to respective tubes, followed by incubation at room temperature for 30 min in the dark. After washing, the appropriate secondary antibody (donkey anti-rabbit Alexa Fluor 488 or rabbit anti-goat Alexa Fluor 647, Abcam) diluted 1:1000 was added (100 µL per sample). Control tubes included samples stained with surface and secondary antibodies only (negative controls). Each sample therefore included a matched negative control (secondary antibody only, without primary antibody), ensuring that the TET protein index (GMFI_test/GMFI_control) inherently corrects for any non-specific binding of the secondary antibody within each gated leukocyte population. Samples were again incubated for 30 min in the dark, washed with Perm/Wash Buffer, centrifuged, and resuspended in 250 µL of PBS for analysis [49].

### 2.5. Data Acquisition and Analysis

Data were acquired using a nine-color CytoFLEX Flow Cytometer (Beckman Coulter, Brea, CA, USA) following daily calibration with CytoFLEX Ready-to-Use QC Fluorospheres (Cat. No. C65719). Analyses were performed using CytExpert 2.4 software (Beckman Coulter).

Cell doublets were excluded based on FSC-A vs. FSC-H gating. Leukocyte populations (granulocytes, monocytes, lymphocytes) were identified by side scatter (SSC-A) and CD45 expression (BV421). Histograms of Alexa Fluor 488-H and Alexa Fluor 647-H fluorescence were used to assess protein expression.

Quantitative results were expressed as the fold change of geometric mean fluorescence intensity (GMFI) in the test sample relative to the corresponding negative control (ratio = GMFI_test/GMFI_control) [50]. Specifically, the TET protein index was calculated as the ratio of GMFI in the test sample (stained with primary and secondary antibodies) to GMFI in the corresponding negative control (stained with secondary antibody only, without primary antibody), measured within the same gated leukocyte population. The geometric mean was used because fluorescence intensity distributions in flow cytometry are log-normal, making it the appropriate measure of central tendency [50]. An index value of 1.0 indicates fluorescence equal to background; values above 1.0 reflect detectable protein expression proportional to the fold increase over non-specific binding. The gating hierarchy and representative histograms for TET1/2/3 expression in each leukocyte subset are provided in Supplementary Figure S1. * *p* < 0.05; ** *p* < 0.01 (Mann–Whitney U test).

The indirect flow cytometry protocol prepared in this manner has been described in detail in the publication by Rożalski et al. [51].

### 2.6. Statistical Analysis

Data are presented as mean ± standard deviation (SD) for normally distributed continuous variables, median (lower quartile; upper quartile) for non-normally distributed (skewed) continuous variables, or counts with percentages (n, %) for categorical variables. Normality was assessed using the Shapiro–Wilk test. Between-group comparisons (statin vs. no statin) were performed using Student’s *t*-test for normally distributed continuous variables or the Mann–Whitney U test for non-normally distributed (skewed) continuous variables, and the χ^2^ (Chi-square) test for categorical variables when expected cell counts were ≥5 in all cells or Fisher’s exact test when any expected cell count was <5. All tests were two-sided, and a *p* value < 0.05 was considered statistically significant. Given the exploratory, hypothesis-generating nature of this pilot study, *p* values were not adjusted for multiple comparisons, and sample size was not determined a priori. To determine the independent effect of statin use on TET expression, we applied multifactorial linear regression analysis. The following factors were used as independent variables: age, gender, blood NT-proBNP, CRP concentration, WBC count, the CHF course (chronic vs. exacerbated), the treatment with statin, MRA, SGLT2, B-blockers. Statistical analyses were conducted using STATISTICA v. 13.1 (TIBCO Software, Inc., Palo Alto, CA, USA, 2017).

### 2.7. Use of Language and Editing Tools

During manuscript preparation, the authors used OpenAI’s ChatGPT (GPT-5.5 Thinking mode) to improve language and readability. All content was reviewed and edited by the authors, who take full responsibility for the scientific accuracy and integrity of the work.

## 3. Results

A total of 106 patients with HFrEF were included in the analysis: 84 were receiving statin therapy and 22 were not. Baseline clinical and laboratory characteristics of both groups are summarised in Table 1. Compared with patients not receiving statins, those on statin therapy had significantly lower plasma NT-proBNP concentrations (2695.6 ± 3574.3 vs. 8050.0 ± 9829.3 pg/mL; *p* < 0.001), lower absolute neutrophil counts (5.8 ± 2.8 vs. 7.8 ± 2.8 g/L; *p* = 0.004), lower lymphocyte counts (2.0 ± 1.0 vs. 3.1 ± 1.6 g/L; *p* < 0.001), and lower monocyte counts (0.8 ± 0.4 vs. 1.0 ± 0.5 g/L; *p* = 0.029). Statin-treated patients also more often received loop diuretics (65.5% vs. 27.3%; *p* = 0.002), mineralocorticoid receptor antagonists (MRAs; 80.9% vs. 36.4%; *p* < 0.001), β-blockers (96.4% vs. 54.6%; *p* < 0.001), sodium–glucose co-transporter-2 (SGLT2) inhibitors (65.4% vs. 36.4%; *p* = 0.016), and aspirin (30.9% vs. 4.6%; *p* = 0.012) (Table 1).

**Figure 2:**
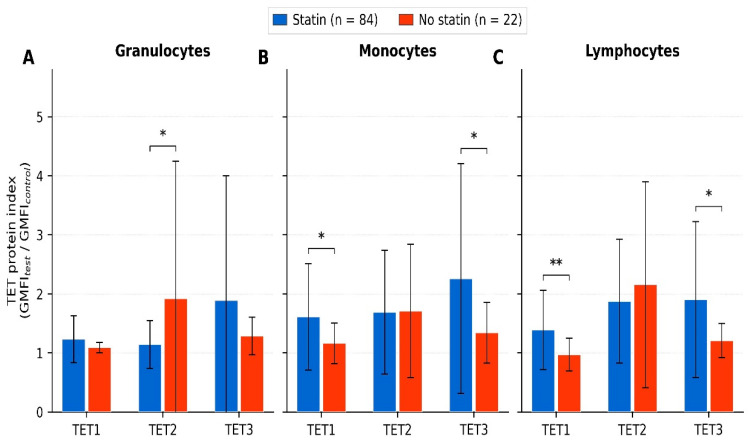
Comparison of the TET protein expression between HFrEF patients treated with and without statins. TET1, TET2 and TET3 protein expression indices in circulating granulocytes (**A**), monocytes (**B**) and lymphocytes (**C**) of HFrEF patients treated with statins (blue; *n* = 84) and without statin therapy (red; *n* = 22). Bars represent mean values; error bars indicate standard deviation. The index is defined as the ratio of geometric mean fluorescence intensity (GMFI) of the test sample to the corresponding negative control. TET1/2/3—Ten–Eleven Translocation methylcytosine dioxygenase 1/2/3; GMFI—geometric mean fluorescence intensity. * *p* < 0.05; ** *p* < 0.01 (Mann–Whitney U test). Numerical values for all TET protein indices are provided in Supplementary Table S1.

With regard to epigenetic markers, statin therapy was associated with a distinct TET expression profile. Patients on statins had significantly higher TET1 indices in monocytes (1.609 ± 0.899 vs. 1.164 ± 0.343; *p* = 0.034) and lymphocytes (1.389 ± 0.669 vs. 0.969 ± 0.278; *p* = 0.008), higher TET3 indices in monocytes (2.256 ± 1.947 vs. 1.343 ± 0.516; *p* = 0.048) and lymphocytes (1.904 ± 1.320 vs. 1.209 ± 0.292; *p* = 0.031), and lower TET2 indices in granulocytes (1.143 ± 0.404 vs. 1.918 ± 2.327; *p* = 0.011), compared with patients not receiving statins (Figure 2).

Next, we performed multifactorial analysis using the multiple regression method in order to determine factors influencing TET protein expression. We found that only G TET2 INDEX was independently determined by treatment course with statin; however, the determination coefficient of regression equation obtained was very low (R^2^ = 0.18; ß = −0.37, *p* = 0.01). Statistically significant multiple regression equations were also obtained for M TET3 INDEX and L TET3 INDEX, but their values were determined independently and positively only by the chronic course of CHF and β-blocker use (only L TET3 INDEX, with negative relationships). Given the low determination coefficients obtained (R^2^ ≤ 0.18) and the ratio of observations to predictors at the lower boundary of recommended thresholds, these regression results should be interpreted with caution and are presented as hypothesis-generating rather than confirmatory.

## 4. Discussion

TET1, TET2 and TET3 are enzymes involved in active DNA demethylation, but they also act as scaffold proteins that integrate different signalling pathways and thereby influence chromatin structure and gene expression from development to immune responses [1,52]. TET1 and TET3 contain CXXC domains and can bind DNA directly, which makes them relatively broad regulators in both developmental and homeostatic contexts [53]. In contrast, TET2 lacks a CXXC domain and appears to act in more specialised settings, particularly in hematopoiesis and control of inflammatory responses [1,22]. This pattern is compatible with an immune-cell TET expression profile aligned with the anti-inflammatory and reparative profile attributed to statins, and the course of disease. Statins themselves inhibit NLRP3 inflammasome activation and IL-1β production [43], so a reduced need for TET2-dependent repression of inflammatory pathways in myeloid cells is a plausible explanation for the lower TET2 indices we observed. Rather than indicating a harmful loss of function, this reduction may reflect an adaptation to a less inflammatory milieu. Although we did not directly analyse inflammasome-related pathways, experimental work suggests that changes in TET2 availability can reshape epigenetic programmes that govern cytokine production and the inflammatory phenotype of circulating monocytes [43,54]. In addition, TET2 is a well-recognised target of a broad network of microRNAs [54], and statin therapy, including atorvastatin and simvastatin, has been shown to alter the expression of several microRNAs in peripheral blood cells [55]. Taken together, these observations are consistent with the idea that statins may modulate TET2 expression in HFrEF through combined effects on inflammation and microRNA networks [43,54,55].

Other immune cell populations may also contribute to the observed TET pattern. TET proteins stabilise anti-inflammatory gene programmes in regulatory T and B cells [53,56], and TET-dependent demethylation of the FOXP3 locus is essential for sustained Treg suppressive activity [56]. TET3 has been implicated in “inflammatory deprogramming” of monocytes, supporting their transition towards a reparative phenotype [34,56]. Statins dampen NF-κB signalling through inhibition of mevalonate synthesis [43], and NF-κB has been reported to directly repress TET1 transcription via p65 binding to the TET1 promoter [52]. Partial suppression of this pathway by statins could therefore contribute to the higher TET1 indices detected in our statin-treated patients [43,52]. Taken together, TET1 and TET3 appear to be engaged less by acute inflammatory triggers and more by signals related to tissue homeostasis and longer-term immune stabilisation [53,56], and the parallel increase in both isoforms may reflect epigenetic consolidation of a less pro-inflammatory immune state.

The borderline differences in LVDd and LVEF between statin-treated and untreated patients (*p* = 0.061 and *p* = 0.053, respectively), together with significantly higher NT-proBNP levels and a greater prevalence of heart failure exacerbations in the no-statin group, suggest that statin-untreated patients may represent a phenotype with more advanced ventricular remodelling. Although representative echocardiographic images were not available for publication, these trends are clinically consistent and warrant investigation in larger cohorts with standardised imaging protocols.

Overall, our findings are consistent with a model in which suppression of chronic inflammation—potentially including effects associated with statin therapy—may reduce the need for strong TET2-mediated restraint of inflammasome-driven cytokine production, while at the same time potentially creating conditions that could favour TET1- and TET3-dependent stabilisation of reparative programmes [43,53,56]. In such a setting, the epigenomic focus may gradually shift from active counter-regulation of inflammatory pathways towards maintenance of differentiation, immune tolerance and vascular homeostasis. This interpretation remains speculative, but it offers a coherent framework that links the opposite directions of TET2 and TET1/TET3 changes observed in our statin-treated patients and can be tested in future mechanistic studies.

Our analysis of TET protein expression in statin-treated and untreated patients with HFrEF provides additional information on epigenetic correlates of statin treatment patterns. The consistent pattern of higher TET1 and TET3 and lower TET2 indices in selected leukocyte subsets suggests that TET proteins may capture aspects of residual inflammatory activity and reparative capacity that are not fully reflected by standard markers such as NT-proBNP or high-sensitivity C-reactive protein. In principle, TET-related indices could therefore become candidates for inclusion in composite biomarker panels aimed at more refined phenotyping and risk stratification in HFrEF.

To the best of our knowledge, reference values for intracellular TET1/2/3 protein abundance measured by flow cytometry in circulating leukocytes have not been established for either healthy individuals or other cardiovascular disease populations. Our study provides the first such dataset in HFrEF, and future work should include healthy age-matched controls to determine whether the observed TET expression profiles are disease-specific or reflect broader population variation.

At the same time, our study has important limitations. It was conducted as a single-centre project with a relatively small sample size and an unequal distribution of patients between the statin and non-statin groups. Statin use was not randomised but based on clinical judgement, so residual confounding by indication cannot be excluded. Moreover, we concentrated on intracellular TET protein levels in circulating leukocytes and did not measure downstream DNA methylation or hydroxymethylation patterns, nor did we systematically assess clonal haematopoiesis.

Although flow cytometry measures fluorescence intensity at the single-cell level within gated populations, which mitigates the confounding effect of between-group differences in absolute cell counts, we cannot exclude that shifts in the proportional representation of subpopulations within each gate (e.g., classical versus non-classical monocytes, or T-cell versus B-cell subsets within the lymphocyte gate) may have contributed to the observed differences in TET indices. Notably, the divergent, isoform-specific directionality of TET changes within the same gated populations—the TET1 and TET3 indices were higher, whereas the TET2 indices were lower, in the statin-treated group—argues against a purely compositional explanation, as a simple shift in subpopulation proportions would be expected to produce concordant, unidirectional changes across all three isoforms. Nevertheless, definitive resolution of cell-intrinsic versus compositional effects would require physical cell sorting (FACS) or high-dimensional computational deconvolution (e.g., FlowSOM, UMAP-based clustering) and should be incorporated in future studies.

Importantly, we did not observe a statistically significant association between TET protein indices and hard clinical endpoints in the follow-up period assessed. Mortality rates were comparable between the statin-treated and untreated groups (20.2% vs. 27.3%; *p* = 0.464), as were hospitalization rates (32.1% vs. 13.6%; *p* = 0.093). Several methodological factors likely account for this finding. First, the cross-sectional design of our study, in which TET protein abundance was measured at a single time point, inherently precludes assessment of the temporal relationship between epigenetic profiles and subsequent clinical events. Landmark studies linking clonal haematopoiesis driven by inactivating TET2 mutations to adverse cardiovascular outcomes employed prospective designs with median follow-up periods of 2.6–4.4 years [25,26,27]. Second, our cohort of 106 patients with an unequal group distribution (84 vs. 22) was underpowered to detect moderate effect sizes for relatively infrequent events such as death; a post hoc estimate indicates that at least 250–300 patients per group would be required to demonstrate a 10-percentage-point difference in mortality with 80% power at α = 0.05. Third, the follow-up period was neither prespecified nor uniform across participants, further limiting the reliability of endpoint ascertainment. Fourth, confounding by indication represents a major concern: statin-treated patients were significantly more likely to receive full guideline-directed medical therapy—including β-blockers (96.4% vs. 54.6%), mineralocorticoid receptor antagonists (80.9% vs. 36.4%), and SGLT2 inhibitors (65.4% vs. 36.4%)—all of which independently reduce mortality and hospitalization in HFrEF [44]. Isolating an independent effect of TET expression on hard endpoints is therefore not feasible in this cohort without propensity score matching or similar methods, which in turn require substantially larger sample sizes. Fifth, it must be emphasised that intracellular TET protein abundance, as measured by flow cytometry, is conceptually distinct from somatic inactivating TET2 mutations underlying clonal haematopoiesis of indeterminate potential (CHIP); the prognostic value of TET protein levels in circulating leukocytes has not been established and requires dedicated validation. Nevertheless, the statin-treated group—which exhibited higher TET1 and TET3 and lower TET2 indices—also demonstrated significantly lower NT-proBNP concentrations (2695.6 vs. 8050.0 pg/mL; *p* < 0.001) and a markedly lower rate of heart failure exacerbations (23.8% vs. 77.3%; *p* < 0.001), both of which are well-validated surrogate markers of disease severity and prognosis in HFrEF. Furthermore, multifactorial regression confirmed that the clinical course (stable vs. exacerbated) independently determined M TET3 and L TET3 expression, supporting a link between TET activity and disease trajectory. Taken together, the absence of a demonstrable correlation with hard endpoints in this pilot study reflects its inherent methodological constraints rather than evidence against a biological association. Prospective validation in a larger, uniformly followed cohort with prespecified endpoints is warranted to determine whether circulating leukocyte TET profiles carry independent prognostic value in HFrEF.

The primary aim of this study was to determine whether differences in intracellular TET1/2/3 protein abundance across leukocyte subpopulations are detectable in HFrEF and whether these protein-level indices relate to inflammatory phenotypes and statin treatment patterns. Importantly, we assessed intracellular TET protein abundance only; we did not measure TET mRNA expression, enzymatic activity, or downstream products of active DNA demethylation/deamination (including 5mC/5hmC dynamics). Therefore, while our observations are biologically plausible and align with prior mechanistic literature, functional validation was beyond the scope of the present study. Future work should extend this framework by integrating transcript-level measurements and direct assessment of DNA (hydroxy)methylation dynamics and related derivatives, ideally in longitudinal designs. Notably, the between-group differences in TET protein levels observed here appear sufficiently pronounced to support stepwise translational validation, and multiparameter flow cytometry provides a single-cell approach to interrogate immune-cell epigenetic markers relevant to inflammatory phenotyping in HFrEF.

Given the number of comparisons performed and the exploratory design of this pilot study, the statistical findings should be interpreted cautiously and considered hypothesis-generating, requiring confirmation in larger, independent cohorts. We also acknowledge that, while statin exposure in the treated group was at least 6 months with a stable type and dose for a minimum of 3 months prior to blood sampling, the exact total duration of therapy could not be reliably quantified from available records; therefore, we were unable to perform dose–duration or duration–response analyses. In addition, TET protein abundance and function may be influenced by multiple biological and environmental determinants that were not systematically assessed in our cohort; therefore, residual confounding cannot be excluded.

The predominance of atorvastatin (64.3%) and rosuvastatin (34.5%) in the statin-treated group, with only one patient receiving simvastatin, precluded meaningful subgroup analysis by statin type or potency; differential epigenetic effects of individual statins cannot therefore be assessed. Moreover, dyslipidaemia itself—independently of statin treatment—has been reported to induce epigenetic alterations in hematopoietic stem and progenitor cells, including modulation of TET1 activity [XX]. Since dyslipidaemia was significantly more prevalent in the statin-treated group (80.9% vs. 18.2%; *p* < 0.001), the observed differences in TET expression may partly reflect the underlying dyslipidaemic state rather than a direct pharmacological effect of statins. This represents an additional confounding factor that cannot be disentangled in the present cross-sectional design [57].

Furthermore, the polyclonal antibodies used for TET detection were not validated in TET-knockout or TET-overexpressing cell lines, and reliance on a single antibody per target without independent confirmation (e.g., by Western blot in the same samples) limits certainty about signal specificity.

Taken together, these constraints—including the absence of functional validation (TET mRNA, enzymatic activity, 5hmC/5mC dynamics), the lack of CHIP screening, the potential for compositional confounding within gated populations, the small and unequally distributed sample, confounding by indication, non-standardised follow-up, and the inability to perform dose–duration analyses—underscore the exploratory, hypothesis-generating nature of the present pilot study and the need for prospective validation in larger, uniformly followed cohorts with prespecified endpoints, physical cell sorting, and integrated assessment of TET mRNA, protein, and enzymatic activity

Further work on the epigenetic actions of statins may help to refine how these drugs are used in cardiovascular medicine and possibly in other fields as well. Statins already provide clinical benefit that extends beyond LDL-cholesterol lowering, and accumulating data support their role as “epidrugs” that influence DNA methylation, histone modifications and non-coding RNA networks [36,37,38,40,41]. Our observations suggest that TET proteins form part of this broader epigenetic footprint in patients with HFrEF, independent of statin use and the course of disease (multifactorial analysis). If confirmed, TET-related pathways might be exploited both as biomarkers and as therapeutic targets to enhance the cardioprotective effects of statins through mechanisms that are at least partly independent of lipid lowering [36,37,38]. From a clinical perspective, our findings have several potential implications. First, if validated in prospective cohorts, TET protein indices in circulating leukocytes could serve as accessible, flow cytometry-based biomarkers that complement established markers such as NT-proBNP and hsCRP for refined inflammatory phenotyping and risk stratification in HFrEF. Second, the association between statin therapy and a distinct TET expression profile adds a new dimension to the understanding of statin pleiotropism, suggesting that epigenetic modulation of immune cells may represent a previously underappreciated component of statin-mediated cardioprotection. Third, the independent association of TET3 indices with the clinical course of heart failure (stable vs. exacerbated) raises the possibility that TET3 protein abundance could be explored as a marker of disease stability. Finally, should the mechanistic link between statins and TET-dependent epigenetic pathways be confirmed, it could open avenues for targeted epidrug strategies aimed at enhancing the non-lipid-lowering benefits of statins in heart failure. However, all of these implications remain speculative at the present stage and require confirmation in adequately powered, prospective studies with functional validation.

## 5. Conclusions

Our findings indicate that, in patients with chronic HFrEF, statin use is associated with a reproducible pattern of TET1/2/3 expression in circulating immune cells, characterised by higher TET1 and TET3 and lower TET2 indices in selected leukocyte subsets. These exploratory data are consistent with the concept that differential TET protein abundance may be associated with statin treatment in HFrEF. However, these findings reflect protein-level associations only, do not establish causality or functional epigenetic reprogramming, and require confirmation in larger, prospective cohorts with integrated functional validation. If confirmed in larger prospective studies, TET-related indices could complement existing biomarkers for inflammatory phenotyping in HFrEF and provide new insights into the epigenetic dimension of statin-mediated cardioprotection.

## Figures and Tables

**Table 1:** Comparison of clinical characteristics between HFrEF patients with and without statin treatment.

Parameter	Statin (*n* = 84)	No Statin (*n* = 22)	*p*-Value
Age (years) ^a^	67.5 ± 12.0	63.2 ± 15.3	0.166
Male, *n* (%) ^b^	63 (75.0)	17 (77.3)	0.764
Hypertension, *n* (%) ^b^	50 (59.5)	10 (45.5)	0.264
Dyslipidaemia, *n* (%) ^b^	68 (80.9)	4 (18.2)	<0.001 *
Diabetes mellitus, *n* (%) ^b^	33 (39.2)	5 (22.7)	0.162
ICM/DCM, *n* (%) ^b^	48 (57.1)	9 (40.9)	0.196
AF/AFL, *n* (%) ^b^	40 (47.6)	8 (36.4)	0.313
AF, *n* (%) ^b^	27 (32.1)	6 (27.3)	0.688
Cardiac pacemaker/CRT-d, *n* (%) ^b^	37 (44.0)/8 (9.5)	3 (13.6)/9 (40.9)	<0.001 *
NT-proBNP (pg/mL) ^a^	2695.6 ± 3574.3	8050.0 ± 9829.3	<0.001 *
WBC (g/L) ^a^	8.1 ± 2.8	7.7 ± 2.2	0.592
HGB (g/dL) ^a^	14.1 ± 1.9	14.5 ± 2.1	0.477
RDW (%) ^a^	14.4 ± 2.0	14.7 ± 1.5	0.510
PLT (g/L) ^a^	215.8 ± 72.3	222.8 ± 72.0	0.689
Neutrophils (%) ^a^	63.3 ± 10.8	62.4 ± 10.6	0.751
Lymphocytes (%) ^a^	23.4 ± 9.7	24.7 ± 9.6	0.558
Monocytes (%) ^a^	8.6 ± 2.8	8.2 ± 3.3	0.592
Neutrophils (g/L) ^a^	5.8 ± 2.8	7.8 ± 2.8	0.004 *
Lymphocytes (g/L) ^a^	2.0 ± 1.0	3.1 ± 1.6	<0.001 *
Monocytes (g/L) ^a^	0.8 ± 0.4	1.0 ± 0.5	0.029 *
Creatinine (mg/dL) ^a^	1.1 ± 0.3	1.1 ± 0.4	0.501
eGFR (ml/min/1.73 m^2^) ^a^	72.2 ± 21.1	71.0 ± 25.1	0.823
Glucose (mg/dL) ^a^	113.3 ± 28.2	114.1 ± 30.8	0.897
hsCRP (mg/L) ^a^	7.4 ± 11.4	12.9 ± 20.1	0.094
LVDd (mm) ^a^	60.5 ± 7.5	64.1 ± 10.1	0.061
LVEF (%) ^a^	29.8 ± 6.8	26.4 ± 9.4	0.053
TAPSE (mm) ^a^	17.8 ± 4.5	16.2 ± 5.2	0.160
Exacerbation of HF, *n* (%) ^b^	20 (23.8)	17 (77.3)	<0.001 *
Hospitalization, *n* (%) ^b^	27 (32.1)	3 (13.6)	0.093
Death, *n* (%) ^c^	17 (20.2)	6 (27.3)	0.464
Loop diuretic, *n* (%) ^b^	55 (65.5)	6 (27.3)	0.002 *
Digoxin, *n* (%) ^c^	6 (7.1)	0 (0)	0.203
Beta-blocker, *n* (%) ^c^	81 (96.4)	12 (54.6)	<0.001 *
SGLT2 inhibitor, *n* (%) ^b^	55 (65.4)	8 (36.4)	0.016 *
ARNI, *n* (%) ^b^	26 (30.9)	3 (13.6)	0.112
ACEi, *n* (%) ^b^	41 (48.8)	8 (36.4)	0.324
Sartan, *n* (%) ^c^	3 (3.5)	2 (9.0)	0.275
MRA, *n* (%) ^b^	68 (80.9)	8 (36.4)	<0.001 *
DOAC, *n* (%) ^b^	33 (39.2)	6 (27.3)	0.320
VKA, *n* (%) ^c^	7 (8.3)	0 (0)	0.167
ASA, *n* (%) ^b^	26 (30.9)	1 (4.6)	0.012 *

* Statistically significant (*p* < 0.05). ^a^ Student’s *t*-test (continuous variables); ^b^ χ^2^ test (categorical variables, all expected counts ≥ 5); ^c^ Fisher’s exact test (categorical variables, any expected count < 5). Continuous variables are presented as mean ± standard deviation; categorical variables as *n* (%). Abbreviations: ICM—ischaemic cardiomyopathy; DCM—dilated cardiomyopathy; AF—atrial fibrillation; AFL—atrial flutter; CRT-d—cardiac resynchronization therapy defibrillator; NT-proBNP—N-terminal pro-B-type natriuretic peptide; WBC—white blood cells; HGB—haemoglobin; RDW—red cell distribution width; PLT—platelets; eGFR—estimated glomerular filtration rate (CKD-EPI); hsCRP—high-sensitivity C-reactive protein; LVDd—left ventricular end-diastolic dimension; LVEF—left ventricular ejection fraction; TAPSE—tricuspid annular plane systolic excursion; HF—heart failure; SGLT2—sodium–glucose co-transporter 2; ARNI—angiotensin receptor–neprilysin inhibitor; ACEi—angiotensin-converting enzyme inhibitor; MRA—mineralocorticoid receptor antagonist; DOAC—direct oral anticoagulant; VKA—vitamin K antagonist; ASA—acetylsalicylic acid.

## Data Availability

The deidentified dataset that supports the findings of this study is not publicly available due to institutional and legal restrictions on sharing individual-level clinical data. The data are available from the corresponding author on reasonable request.

## Supplementary Materials

The following supporting information can be downloaded at: https://www.mdpi.com/article/10.3390/cimb48050467/s1.
