# Supplementary material for: Differential TET1/2/3 Protein Expression in Circulating Leukocytes of Statin-Treated Patients with HFrEF
Source: Curr Issues Mol Biol. 2026 Apr 30;48(5):467. doi: 10.3390/cimb48050467 (PMC13204469; doi:10.3390/cimb48050467)
Supplement: Supplementary file 1 [file cimb-48-00467-s001.zip › cimb-4211589-supplementary.pdf]

Supplementary Table S1. Comparison of the TET protein expression between HFrEF patients treated with and without statins.

Parameter	Statin (n = 84)	No Statin (n=22)	p value
G TET1 INDEX	1.230 ± 0.398	1.090 ± 0.089	0.135
G TET2 INDEX	1.143 ± 0.404	1.918 ± 2.327	0.011
G TET3 INDEX	1.889 ± 2.111	1.284 ± 0.318	0.245
M TET1 INDEX	1.609 ± 0.899	1.164 ± 0.343	0.034
M M TET2 INDEX	1.689 ± 1.049	1.711 ± 1.128	0.936
M M TET3 INDEX	2.256 ± 1.947	1.343 ± 0.516	0.048
L INDEX TET1	1.389 ± 0.669	0.969 ± 0.278	0.008
L TET2 INDEX	1.875 ± 1.050	2.155 ± 1.745	0.377
L TET3 INDEX	1.904 ± 1.320	1.209 ± 0.292	0.031

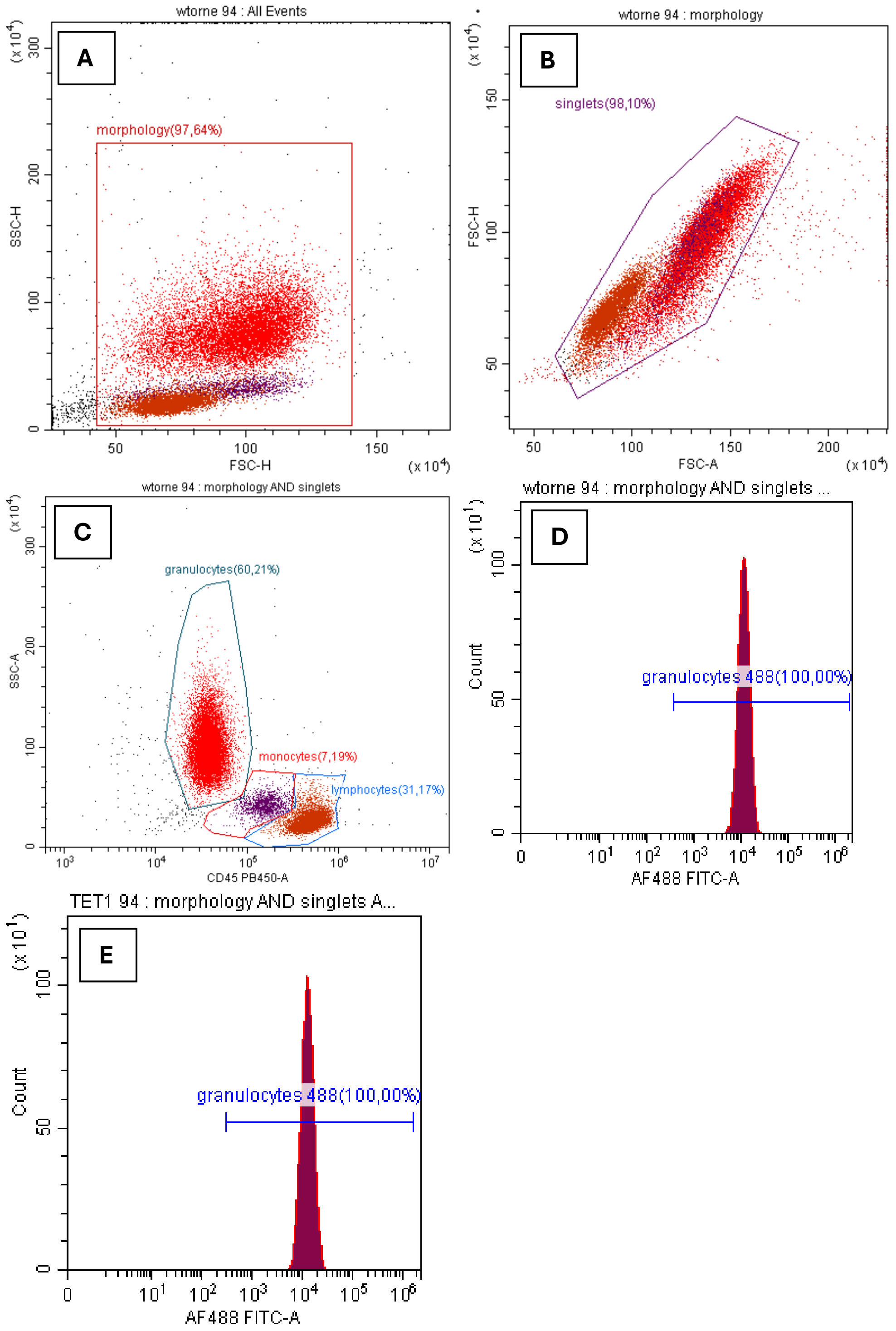

Supplementary Figure S1. Cytometric analysis - gating strategy and determination of enzyme protein expression levels in peripheral blood cell populations in patients with heart failure with reduced ejection fraction: A-morphology, B-dot plot showing gating of cell populations with rejection of doublets. C- Differentiation of cell populations based on the leukocyte marker CD45 and the SSC parameter determining cell granularity. D- Histogram showing background (control only with fluorochrome conjugated antibody) E- Histogram showing test sample with antibody directed against the test protein (anti-TET-1, anti-rabbit Alexa Fluor 488).
